# An Analysis of Proteochemometric and Conformal Prediction Machine Learning Protein-Ligand Binding Affinity Models

**DOI:** 10.3389/fmolb.2020.00093

**Published:** 2020-06-24

**Authors:** Conor Parks, Zied Gaieb, Rommie E. Amaro

**Affiliations:** Department of Chemistry and Biochemistry, University of California, San Diego, La Jolla, CA, United States

**Keywords:** conformal prediction, proteochemometric, protein-ligand binding affinity, bemis-murcko scaffolding, random forest, deep neural net (DNN)

## Abstract

Protein-ligand binding affinity is a key pharmacodynamic endpoint in drug discovery. Sole reliance on experimental design, make, and test cycles is costly and time consuming, providing an opportunity for computational methods to assist. Herein, we present results comparing random forest and feed-forward neural network proteochemometric models for their ability to predict pIC50 measurements for held out generic Bemis-Murcko scaffolds. In addition, we assess the ability of conformal prediction to provide calibrated prediction intervals in both a retrospective and semi-prospective test using the recently released Grand Challenge 4 data set as an external test set. In total, random forest and deep neural network proteochemometric models show quality retrospective performance but suffer in the semi-prospective setting. However, the conformal predictor prediction intervals prove to be well-calibrated both retrospectively and semi-prospectively showing that they can be used to guide hit discovery and lead optimization campaigns.

## Introduction

One of the most important phases of a drug discovery campaign is the discovery of a potent inhibitor to a target driving the disease phenotype. Experimental design, make, test cycles seek to optimize initial hits to lead compounds by optimizing the protein-ligand binding affinity. However, this process is frequently slow and costly, adding to the large cost of drug discovery. As such, computational methods that can accelerate this optimization phase by predicting protein-ligand binding affinity values are readily sought. Fully atomistic simulation approaches model protein-ligand binding physics through time integrating Newton's equations of motion in molecular dynamics simulations (Jorgensen and Thomas, 2008; Chodera et al., 2011; Mobley and Klimovich, 2012; Christ and Fox, 2014; Abel et al., 2017; Cournia et al., 2017; Mobley and Gilson, 2017). However, molecular dynamics approaches can suffer from large computational costs, insufficient sampling, and variably accurate force fields. As an alternative, quantitative structure activity modeling (QSAR) uses machine learning (ML) as a stand in for physically rigorous simulations by seeking to model statistical correlations between ligand information and protein-ligand binding affinity (Cherkasov et al., 2014). Traditional QSAR models do not model the protein directly, and hence do not allow learning from related protein family members during training. In contrast proteochemometric (PCM) models combine both protein and ligand information to create a composite feature vector that allows the model to learn mappings between all protein-ligand pairs in a training set (Cortés-Ciriano et al., 2015a). PCM models have been applied to a diverse number of protein families including G-protein coupled receptors (Gao et al., 2013), HDACs (Tresadern et al., 2017), kinases (Subramanian et al., 2013), Cytochrome P450s, HIV proteases (Lapins et al., 2008), Poly(ADP-ribose) polymerases (Cortés-Ciriano et al., 2015b), and bromodomains (Giblin et al., 2018). Recently, PCM and multi-task neural networks have also been benchmarked using ChEMBL data where the utility of PCM modeling for binder/non-binder classification was demonstrated (Lenselink et al., 2017).

We first compare the performance of random forest (RF) and feed-forward neural network (FFN) PCM models trained using the recently released ChEMBL25 data set (Gaulton et al., 2017) with full length protein sequence features on a generic Bemis-Murcko scaffold split. It is shown that the RF and FFN models achieve comparable overall performance suggesting that both models approach the upper levels of performance possible given the heterogeneous IC50 measurements in ChEMBL25 (Kalliokoski et al., 2013). Feature analysis shows that both models leverage the protein sequence features extensively, albeit in different ways. We demonstrate that the optimization of entity embeddings for ECFP6 categorical variables allows FFN models to perform feature engineering in a data driven manner (Guo and Berkhahn, 2016). In addition, we compare the validity and efficiency of regression conformal predictors first in a retrospective test using ChEMBL25 data and subsequently in semi-prospective test using the recent Drug Design Data (D3R) Grand Challenge 4 (GC4) data set (Parks et al., 2019a). The D3R issues blinded prediction challenges to the computer aided drug design (CADD) community to assess method performance in truly blinded scenarios. Although the data has been since released to the community, we use the data in the most recent GC4 (Parks et al., 2019a) data set as a test set external to the data in ChEMBL25 for what we refer to as a semi-prospective test. These results show that the performance of both models suffer on the GC4 dataset relative to the ChEMBL25 validation set. However, the performance is in line with the top performing ML models in prior D3R Grand Challenges (Gathiaka et al., 2016; Gaieb et al., 2018, 2019; Parks et al., 2019a). Finally, the prediction intervals from the conformal predictors are shown to be valid on the GC4 data set, demonstrating the validity of conformal prediction confidence intervals on a high-quality external test set.

## Materials and Methods

### Data Set Source and Preparation

The recently released ChEMBL25 (Gaulton et al., 2017) database was used for ML model training. Only molecules with specified canonical SMILES strings, standard units of nM, no potential duplicates, confidence score of 9, activity comment not equal to inconclusive, and against protein targets with specified gene ids, and protein sequences from Swiss-Prot were kept. Ligands with PAINS patterns identified via RDKit^1^ were removed. Only ligands with a molecular weight in the range of 75 to 800 Da were retained. We replaced multiple IC50 values for the same protein-ligand pair by the median IC50 value. SMILES strings were standardized and canonicalized using the charge parent function in MolVS^2^. SMILES strings were either featurized using 4,096 bit length ECFP6 fingerprints, molecular weight, topological surface area, number of hydrogen donors, number of hydrogen acceptors, LogP, heavy atom count, number of rotatable bonds, and ring count with RDKit or CDDD descriptors (Winter et al., 2019). Protein sequences were featurized using amino acid, dipeptide, composition, transition, and distribution descriptors. Ligand and protein descriptors were then concatenated to create the full feature vector. Non ECFP6 bit values were scaled using the standard scaler function in Scikit-learn (Pedregosa et al., 2011). All IC50 values were converted to pIC50 values and scaled using the standard scaler function. The final data set contained 302,325 data points consisting of 213,502 unique SMILES strings across 940 unique UniProt IDs.

### Machine Learning Model Training and Conformal Prediction

RF models were trained using the Scikit-learn (Pedregosa et al., 2011) library via a grid search hyperparameter optimization strategy. The following hyperparameter values were explored: 100, 500, and 1,000 for the number of estimators; sqrt, log2, 0.3, and 0.5 for max features; and 1,3,5,10,25 for min samples leaf. FFN models were trained using Fast.ai (Howard and Gugger, 2020). We investigated treating ECFP6 bit vectors as categorical variables whose embeddings were optimized via backpropagation during model training (Guo and Berkhahn, 2016). A 3-layer model was employed with 2,000 nodes in the first layer, 1,000 in the second, and 500 in the third. Linear layer outputs were then passed through an activation and then a batch norm and dropout layer. A ReLU function were used for activations, with the exception of the last layer, where a Sigmoid function was used. This was done to facilitate training by scaling outputs from the last linear layer to a range of values between the max and minimum scaled pIC50 values in the training set multiplied by a scaling factor of 1.2. Weight decay was set to 0.01. Dropout of 0.25 was used in each layer, except for the embedding layers, where a dropout of 0.01 was used. All other Fast.ai tabular model defaults were used. The FFN model was trained with the fit_one_cycle (Smith, 2018) method. All models were then analyzed using mean squared error (MSE), Pearson correlation coefficient, and Kendall's Tau metrics.

Rigorous quantification of model confidence is essential in fields such as drug discovery where chemical space is essentially infinitely vast and models are trained on only a small fraction of possible compounds. Conformal prediction is a state of the art method to provide confidence intervals, i.e., a region where the true value is predicted to be, and whose size is determined in part by a user defined confidence level (Shafer and Vovk, 2008; Norinder et al., 2014; Cortés-Ciriano et al., 2015a; Sun et al., 2017; Svensson et al., 2017). Here, we define validity as the frequency at which the confidence interval contains the true value. For example, the 95% confidence prediction intervals for a well-calibrated conformal predictor would contain the true value 95% of the time. The efficiency of a conformal predictor is a reflection of the size of the confidence internals produced. Here, a model that produces smaller confidence intervals than another model would be considered to have higher efficiency. These two variables, validity and efficiency, quantify the performance of conformal prediction models. Conformal prediction requires a way to gauge the similarity of a new piece of data to training data. Recent literature has shown that the standard deviation across the trees of a RF model (Svensson et al., 2018), and the use of test-time dropout in the case of FFN models (Cortés-Ciriano and Bender, 2019a), provide valid and efficient conformal predictors. However, these methods have not been analyzed in prospective settings extensively. We use these methods to assess non-conformity herein. For an overview of conformal prediction in the field of drug discovery, we direct the reader to a recent review (Cortés-Ciriano and Bender, 2019b).

It has been shown that overly simplistic (i.e., random) training/validation/test splits lead to overestimates in the accuracy of machine learning models (Wallach and Heifets, 2018). This is one explanation for the discrepancy between performance metrics seen in retrospective settings and those seen in true prospective tests (Gathiaka et al., 2016; Gaieb et al., 2018, 2019; Parks et al., 2019a). In an attempt to mitigate this, we elected to perform hyperparameter optimization using an 80/20 generic Bemis-Murcko scaffold split. This leads to a more difficult split, as the validation set does not contain any compounds with a generic Bemis-Murcko scaffold already present in the training set. Optimal hyperparameters and performance metrics on the ChEMBL25 data set for all models were first determined with this split. In all subsequent models, these sets of hyperparameters were using for training. The training set was then further divided using a random 80/20 split into a new training and calibration set. Models were then retrained and the calibration set was used to calibrate the confidence intervals of the conformal predictors. These models were used to assess the validity and efficiency of the conformal predictors on the ChEMBL25 validation data set. Finally, the entire ChEMBL25 dataset was randomly split into another 80/20 training and calibration set. All models were again trained and calibrated. These final models were used to assess performance on the external GC4 dataset in a semi-prospective test.

## Results

### Retrospective Analysis

In Table 1 we provide the performance metrics and model architectures for the best performing models across the full validation set.

**Table 1:** ChEMBL25 validation set performance metrics for both the RF and FFN models as well as the SMILES featurization method used.

**Model type**	**MSE (scaled pIC50)**	**Pearson correlation**	**Kendall's Tau**
RF (ECFP6)	0.38	0.80	0.61
RF (CDDD)	0.47	0.75	0.55
FFN (entity embeddings)	0.39	0.79	0.60
FFN (ECFP6)	0.41	0.78	0.58
FFN (CDDD)	0.42	0.78	0.58

Table 1 demonstrates that both model types (RF/FFN) perform equally well on the applied generic Bemis-Murcko scaffold split. We found the RF model with ECFP6 fingerprints, and the FFN model with entity embeddings to be the best performing models. The following sets of hyperparameters were found to be optimal for the RF models: {n_estimators=1000, max_features=sqrt, min_samples_leaf=1} using ECFP6 fingerprints, and {n_estimators=1000, max_features=log2, min_samples_leaf=1} using CDDD features. The scaled pIC50 MSE values in Table 1 translate to a root mean squared error of approximately 0.8 pIC50 units. These values are in the range of expected errors for ML models trained on heterogeneous ChEMBL25 data (Kalliokoski et al., 2013), and are in agreement with prior literature that also demonstrated that RF and FFN models approached the upper limit of overall accuracy across the dataset, given the heterogeneous IC50 measurements in ChEMBL25 (Cortés-Ciriano and Bender, 2019a).

Ensemble averaging is a strategy to improve prediction performance by averaging the individual predictions of multiple models. This wisdom of crowd approach works best when individual models are uncorrelated, allowing errors to be averaged out. The residuals of the RF and FFN model are correlated with an *R*^2^ metric of 0.88. As such, only a modest improvement at best is noted when the FFN and RF model predictions are averaged to yield the following performance metrics: 0.36 MSE, 0.81 Pearson correlation, and 0.61 Kendall's Tau.

The number of data points per gene is heterogeneous in ChEMBL25, allowing a few genes to contribute more to performance metrics than others. To remove this bias, we calculated performance metrics across each individual gene with more than 100 data points in the validation set (Table 2). Analysis of Table 2 shows that model performances vary moderately across the various genes with metric fluctuations (standard deviation/value) on the order of 30–50%. We found the amount of training data for a given UniProt ID to be a poor predictor for future successful predictions. We calculated the Kendall's Tau correlation between amount of data in the training set and the MSEs for each gene and found no correlation (tau = 0.05 both the RF and FFN model). Performance is spread across protein families as well, with the top 30 performing UniProt IDs for the RF model containing proteins from the kinase, protease, and RNA polymerase families. This suggests that chemical similarity and bias between the training and validation set are the most import variables determining performance.

**Table 2:** Performance metrics averaged across individual UniProt IDs.

**Model type**	**MSE (scaled pIC50)**	**Pearson correlation**	**Kendall's Tau**
RF	0.37 +/– 0.17	0.65 +/– 0.18	0.46 +/– 0.15
FFN	0.38 +/– 0.15	0.61 +/– 0.22	0.43 +/– 0.17

**Figure 1:**
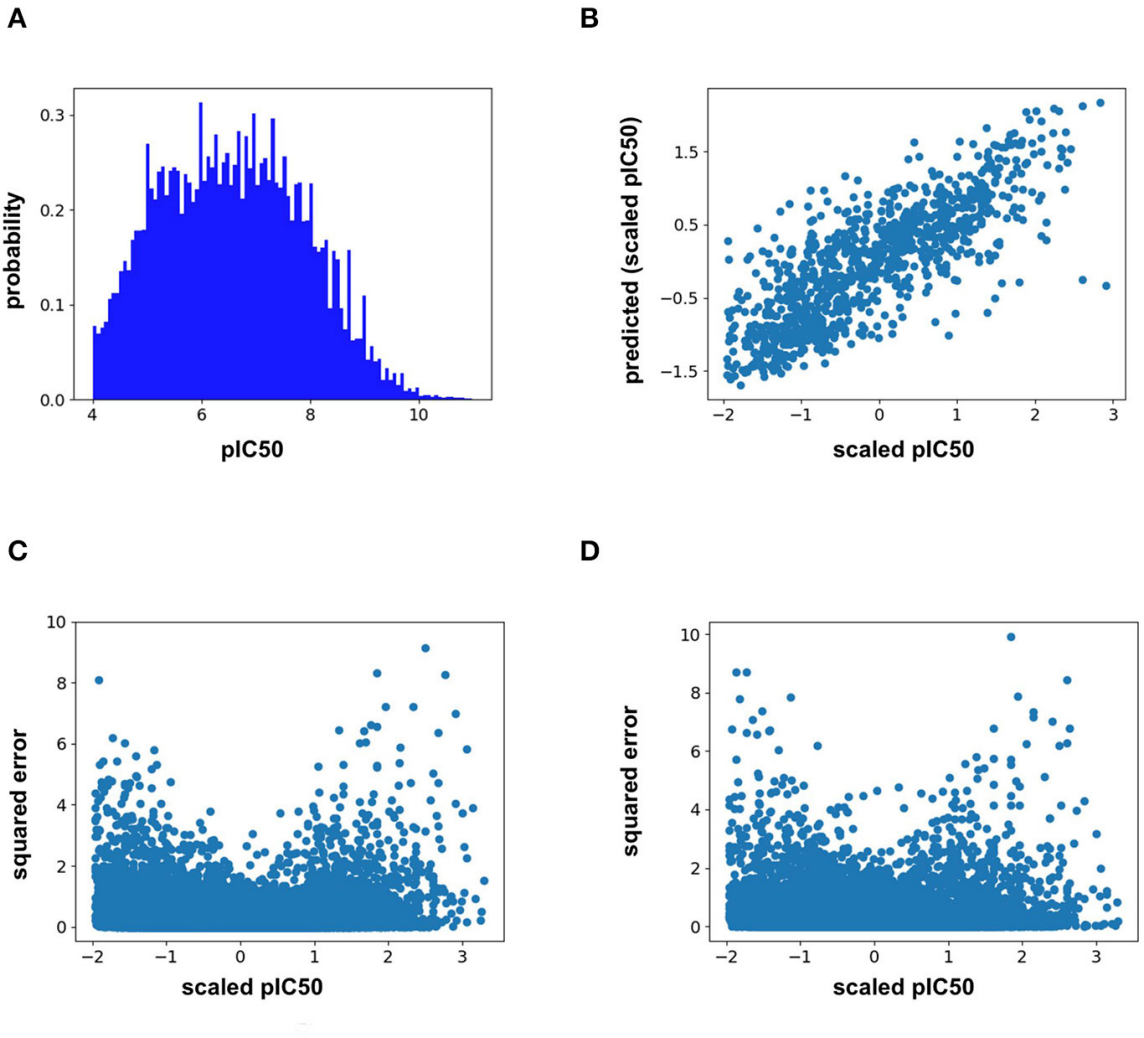
**(A)** ChEMBL25 pIC50 probability distribution, **(B)** validation set scaled pIC50 vs. RF predictions, **(C)** validation set scaled pIC50 vs. RF prediction squared error, and **(D)** validation set scaled pIC50 vs. FFN prediction squared error.

Analysis of the training distribution of the pIC50 values shows that a strong amount of publication bias exists in the ChEMBL25 dataset (Figure 1A). Active compounds are over represented as shown by the average pIC50 value of the whole distribution being 6.57. In true prospective unbiased chemical library screens, it is common for 95% of the compounds to have pIC50 <5 (i.e., non-binders). This demonstrates that the pIC50 predictions from the ML models herein should be used with caution for prospective virtual screening of chemical libraries, as large test-time distribution shift is certain. In addition, the distribution of training pIC50s is non-uniform (Figure 1A). This can lead to heterogeneous model performance across differing ranges of pIC50 values. For example, the RF model predictions correlate well with the measured values for the given split overall (Figure 1B). However, Figures 1C,D demonstrates the accuracy of both the RF and FFN models suffer in the tail of the pIC50 distribution, with quality performance obtained in a range of +/– 1 standard deviation from the mean.

**Figure 2:**
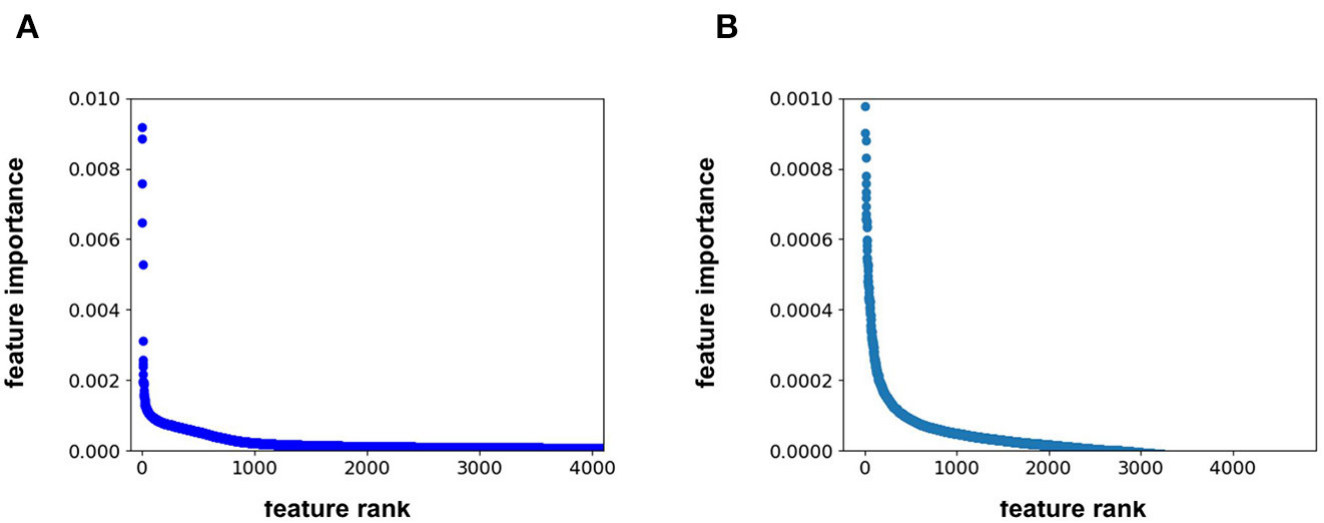
**(A)** feature rank vs. feature importance for the **(A)** RF model and **(B)** FFN model. In **(B)**, the y-axis was capped at 0.001 to maintain resolution.

Feature importance analysis allows us to determine whether the protein sequence component of the feature vector is pertinent to the model performance. This analysis indicates that the feature importance for the RF model plateaus at approximately the 1500th ranked feature out of the total 4,104 features (Figure 2A). All 567 protein sequence features, and all 8 physiochemical property features, fall in the top 1500 features. For the FFN model, the feature importance plateaus at approximately the 2000th ranked feature out of the total 4,104 features for the FFN model (Figure 2B). The top 2,000 features consist of 268 of 567 protein sequence features, 1,725 of 4,096 Morgan fingerprint bits, and 7 of 8 physiochemical properties. This demonstrates that both model types rank protein sequence features among the most important features. Both models find the physiochemical properties features to be the most import features overall, including MW. The Kendall's Tau ranking of the validation set scaled pIC50 values using solely MW alone is 0.15 demonstrating the trend of lead optimization campaigns to result in increasingly larger MW molecules. To further interrogate the protein sequence features, new training and validation sets were generated via a random UniProt ID split and models were retrained. This resulted in a much tougher split with the FFNs being the clearly best performing model by MSE (Table 3). This suggests that the FFN models are able to leverage the protein sequence features more effectively.

**Table 3:** ChEMBL25 validation set performance metrics for both the RF and FFN models as well as the SMILES featurization method used.

**Model type**	**MSE (scaled pIC50)**	**Pearson correlation**	**Kendall's Tau**
RF (ECFP6)	0.96	0.41	0.27
RF (CDDD)	0.89	0.46	0.31
FFN (entity embeddings)	0.80	0.51	0.35
FFN (ECFP6)	0.79	0.49	0.34
FFN (CDDD)	0.78	0.48	0.33

**Figure 3:**
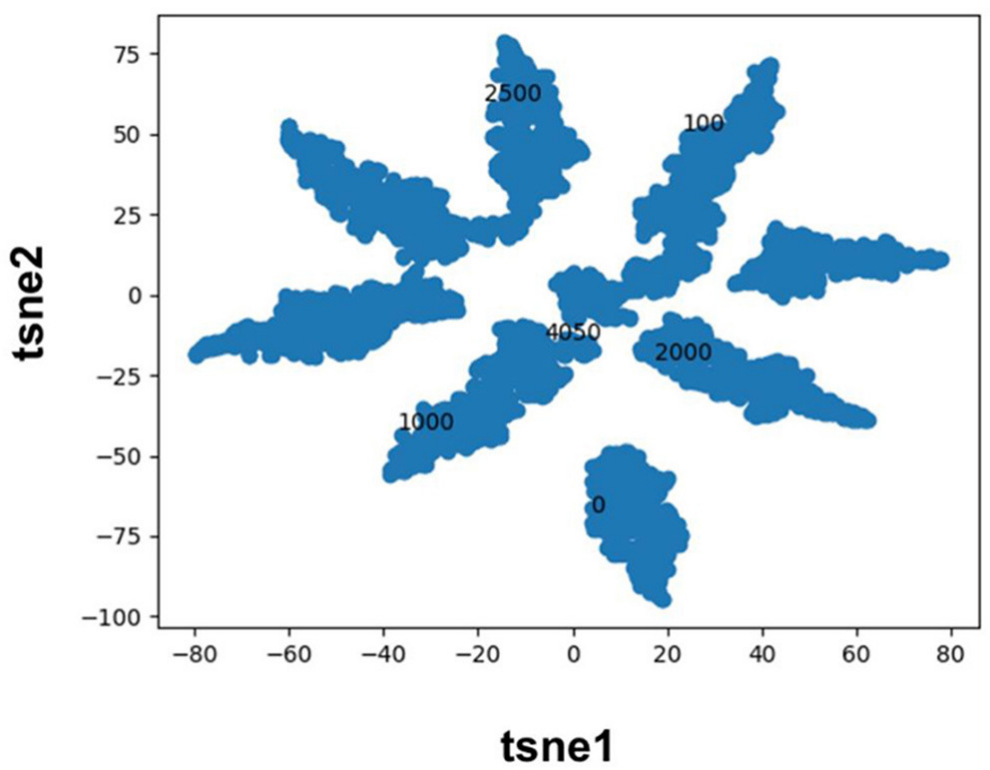
TSNE plot of the FFN entity embeddings for the category 1 variables of the morgan fingerprint vector. Category 1 was selected for plotting as this denotes the presence of a chemical fragment. The category 1 weights of the 0, 100, 1,000, 2,000, 2,500, and 4,050 bits are plotted in black to illustrate how the FFN model groups bits into distinct clusters.

Molecular fingerprints are the most commonly used technique to encode molecules for ML model training. This technique hashes atomic neighborhoods for each atom to bits to represent molecules with 1D dimensional vectors. This approach can suffer from bit collision. In addition, there is no meaning of proximity of bits (Feinberg et al., 2019). As an alternative, prior literature has sought to extract features directly from images (Ragoza et al., 2017; Ciriano and Bender, 2018; Jiménez et al., 2018; Parks et al., 2019b) or graphs (Kearnes et al., 2016; Kipf and Welling, 2017; Feinberg et al., 2019; Torng and Altman, 2019) using convolution neural networks. Here, we pursue a complementary approach where we map categorical variables into Euclidean space using entity embeddings (Guo and Berkhahn, 2016). Entity embeddings are sets of weights in the neural network that represent possible categories of a feature. These weights are then optimized by the neural network during training. Hence, each category (0/1) of each bit in the Morgan fingerprint is now represented by a unique set of weights in the neural network. This allows the network to learn relationships between chemical fragments in a data driven manner. Our results demonstrate that the FFN learns to group Morgan fingerprint bits into multiple clusters through the optimization of embedding weights (Figure 3) providing a novel compound fingerprint. For the generic Murcko scaffold split used herein, the use of entity embeddings led to a 5% reduction in MSE for the FFN model. However, no performance variation was seen between the CDDD/ECFP6/and entity embedding features for the FFN models on the random protein split. Future work will be needed to interrogate the utility of the entity embeddings as a supplement to the tradition ECFP fingerprint.

**Figure 4:**
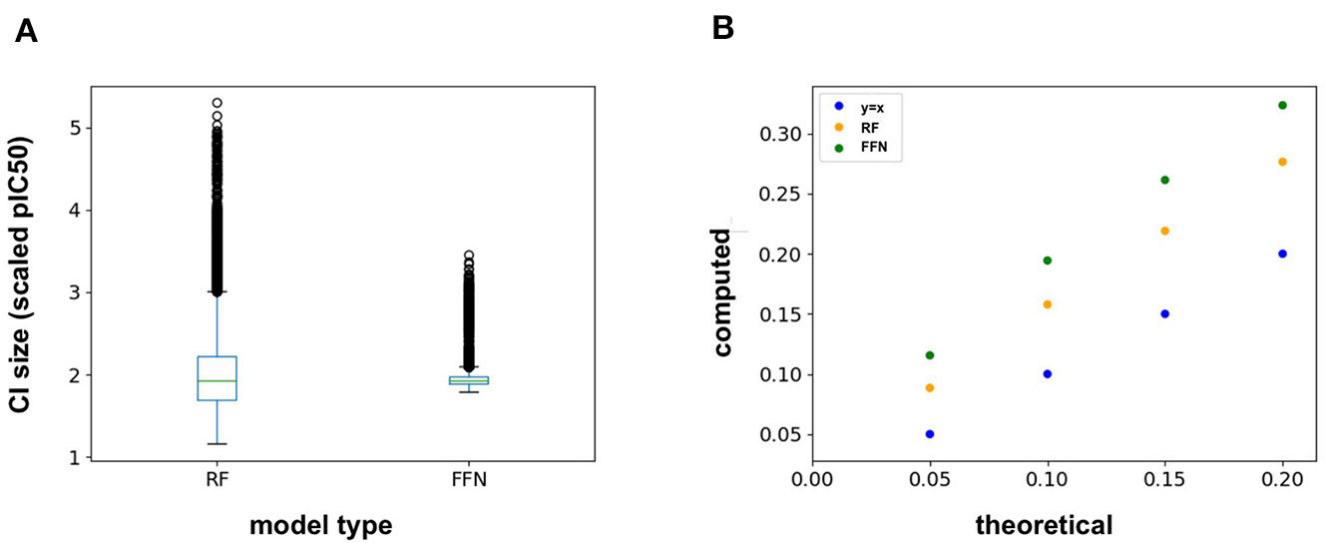
**(A)** Box-whisker plot of the 95% confidence interval (CI) sizes for both the RF and FFN model computed on the validation set, and **(B)** theoretical vs. computed error rates (1-validities) on the validation set for varying error thresholds (1-confidence threshold).

The ChEMBL25 validation set was also used to assess the retrospective performance of conformal prediction. Analysis of the RF prediction interval sizes shows that they span a larger range of values than those from the FFN (Figure 4A), and hence are less efficient. However, the RF model has better validities than the FFN model (Figure 4B), but both models still achieve quality validities overall. Interestingly, there is very little variation in the size of the FFN confidence intervals across all predictions on the validation set (Figure 4A) but this is still sufficient for the FFN to generate valid prediction intervals (Figure 4B). In total, conformal prediction is able to accurately gauge both RF and FFN model confidence for predictions on held-out validation data, in agreement with prior literature (Svensson et al., 2018; Cortés-Ciriano and Bender, 2019a).

### Semi-prospective Test

The true test of model performance is a prospective one as this is how ML models are used in practice. Here, we use the recently released GC4 dataset (Parks et al., 2019a) as an external, semi-prospective test for the best performing models [RF (ECFP6) and FFN (entity embeddings)] from the generic Murcko split. GC4 provided IC50 data to two targets: Cathepsin S (CatS), and Beta-amyloid secretase 1 (BACE-1). Analysis of Table 4 shows that the ranking of model performances is target dependent, with the FFN performing best for BACE-1 and the RF model performing best for CatS. By comparing the metrics for both models on the same targets in the retrospective validation set, we see that both models suffer heavily in the prospective setting (Table 4). This is in agreement with prior literature showing data set split bias inflates model performance metrics (Wallach and Heifets, 2018), despite our attempts to mitigate this via a generic Bemis-Murcko scaffold split. However, these metrics are in line with the performance seen during GC4 with ML models (Parks et al., 2019a). Based on the Kendall's Tau value of the RF model for the CatS dataset, the RF model would have ranked in the top 10 performing methods for the CatS affinity prediction challenge. Similarly, the FFN would have placed in the top 10 for the BACE-1 affinity prediction challenge.

**Table 4:** GC4 BACE-1 and CatS performance metrics.

**Model**	**BACE-1 Pearson correlation**	**BACE-1 Kendall's Tau**	**CatS Pearson correlation**	**CatS Kendall's Tau**
RF	0.25 (0.71)	0.19 (0.51)	0.25 (0.68)	0.38 (0.49)
FFN	0.5 (0.77)	0.29 (0.59)	0.19 (0.8)	0.26 (0.61)

*The corresponding performance metrics on the ChEMBL25 validation sets for BACE-1 and CatS are contained in parenthesis for comparison*.

**Figure 5:**
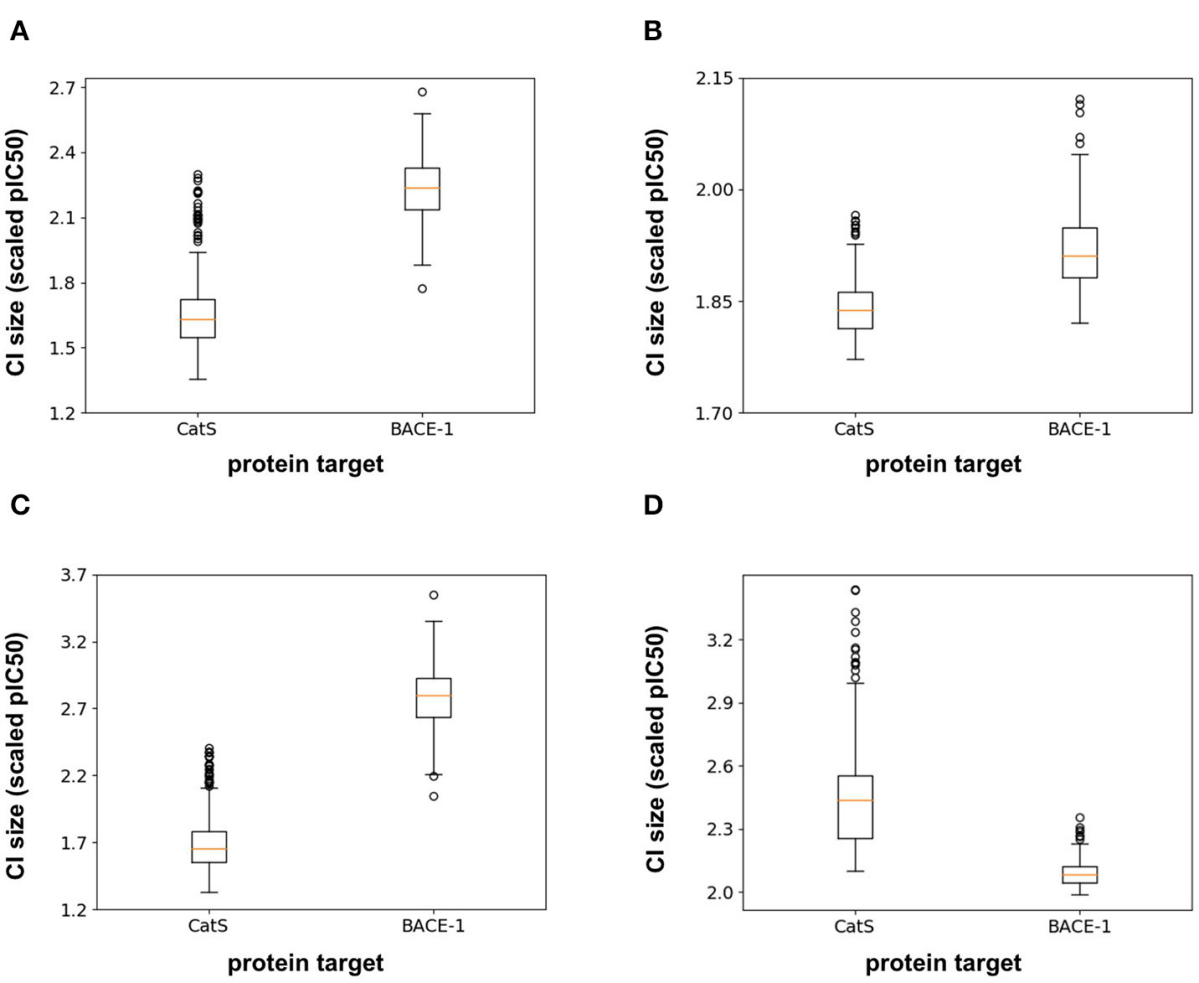
Box-whisker plots of the 95% confidence interval (CI) sizes (scaled pIC50) for CatS/BACE-1 predictions for **(A)** RF PCM model, **(B)** PCM FFN model, **(C)** QSAR RF model, and **(D)** QSAR FFN model.

In addition to the model predictions, this semi-prospective test allows us to analyze the validity and efficiency of the conformal prediction intervals for both the RF and FFN. Both models achieve excellent validities on the CatS data set. Here, the prediction intervals contained the true measured value 95 and 97% of the time for the RF/FFN models, respectively. The validities of both models suffer slightly on the BACE-1 data set relative to CatS, but overall still achieve excellent performance with 81 and 80% validities. As a possible explanation for the validity performance degradation between CatS and BACE1, we note that the distribution of nearest neighbor Tanimoto coefficients demonstrates that the CatS GC4 compounds are more similar to the ChEMBL25 training data, and hence providing an easier test for the conformal predictor, than the BACE1 GC4 compounds (Supplementary Figure 1). For both CatS and BACE-1, we find the FFN provides more efficient prediction intervals (Figures 5A,B). In total, these results demonstrate the ability of the conformal predictors to generate valid prediction intervals in a semi-prospective setting.

We next sought to compare the performance of the PCM models against a standard QSAR target model trained using only CatS or BACE-1 ChEMBL25 data, respectively. Here, a RF QSAR model achieved approximately the same performance metrics on both targets as the RF PCM model with a 0.26 Pearson Correlation and 0.37 Kendall's Tau for CatS and 0.26 Pearson Correlation and 0.18 Kendall's Tau for BACE-1. This indicates that the RF model may benefit from additional protein sequence descriptors such as those from unsupervised training (Kim et al., 2019). The conformal prediction validities remained approximately equivalent at 93% on the CatS data set and 83% on the BACE-1 dataset. However, the data augmentation from PCM training improved the efficiencies of the confidence intervals relative to those from the QSAR RF model for BACE-1 (Figures 5A,C). The FFN QSAR model performance degraded relative to the PCM model with a 0.23 Pearson Correlation and 0.25 Kendall's Tau for CatS and a 0.46 Pearson Correlation and 0.22 Kendall's Tau for BACE-1. This suggests that the FFN is able to leverage the information of other protein sequences in the data set more effectively than the RF model during training. The validities of the FFN QSAR conformal predictor for BACE-1 degraded to 74%, but remained roughly the same at 98% for CatS. We find the PCM FFN model confidence interval to be more efficient than those of the QSAR FFN model (Figures 5B,D). This is most vividly captured in the case of CatS.

Finally, we sought to test the impact of deleting all CatS and BACE1 data and retraining the models using the same procedure. As shown in Supplementary Table 1, this had the expected negative impact on performance metrics for both model types with the FFN performance suffering the least. The only exception was the Pearson's correlation between FFN model predictions and target values for CatS where the metric remained statistically equivalent. Notably, the Kendall's Tau ranking of model predictions is now only as good as a LogP ranking of the data for both model types. However, the conformal predictor remains well-calibrated with a 99 and 80% validity for the RF model, and 80 and 72% validity for the FFN model for CatS and BACE1, respectively. Supplementary Figure 2 demonstrates that there is a marked reduction in the efficiency of the prediction intervals from the RF model. However, the FFN prediction interval efficiencies only degrade slightly. Despite the reduction in the performance of the point prediction values, we conclude conformal prediction remains an impactful method to gauge model confidence even on never before seen protein targets.

## Conclusion

Protein-ligand binding affinity is a key variable during hit discovery and lead optimization in drug discovery. Experimental design, make, test cycles that seek to optimize this property are costly and time consuming, and hence limit the rate of entry of novel therapies to the clinic. Computational methods seek to accelerate these cycles by producing reliable protein-ligand binding affinity predictions. Traditional QSAR models train using only chemical compound data for a specific target. Alternatively, PCM models featurize both protein sequence and ligand to create the final feature vector. This allows ML models to be trained on protein-ligand binding affinity data from multiple proteins at once, hence augmenting the size of the training set, and potentially allowing the model to learn from related proteins (Lapins et al., 2008; Gao et al., 2013; Subramanian et al., 2013; Cortés-Ciriano et al., 2015a,b; Tresadern et al., 2017; Giblin et al., 2018).

Here we first analyze the performance of PCM models trained using the most recent ChEMBL25 database (Gaulton et al., 2017). The results above show that a RF and FFN model achieve comparable performance on the generic Bemis-Murcko scaffold split of ChEMBL25 data. The root mean squared error of the models were approximately 0.8 pIC50 units suggesting both models are approaching the limit of accuracy given the heterogenous IC50 measurements in ChEMBL25 (Kalliokoski et al., 2013). Feature importance analysis of both models demonstrated that protein sequence features were among the most important features overall. We show that entity embeddings for the categorical ECFP6 Morgan fingerprints can be optimized during FFN training and provide quality performance for drug discovery applications (Guo and Berkhahn, 2016). This allows for feature engineering in a data driven manner and provides an alternative to other methods that seek to derive novel chemical features using convolutional (Ragoza et al., 2017; Ciriano and Bender, 2018; Jiménez et al., 2018; Parks et al., 2019b) and graph-convolutional neural networks (Kearnes et al., 2016; Kipf and Welling, 2017; Torng and Altman, 2019).

Finally, we analyze the utility of conformal prediction to provide prediction intervals to assess model confidence. Conformal prediction was implemented using the standard deviation across the trees of the RF model and Bayesian dropout (Cortés-Ciriano and Bender, 2019a) for the FFN model. Both models generated well-calibrated and efficient confidence intervals on the ChEMBL25 validation set. In addition, we assessed the performance of the RF model in a semi-prospective setting using the recently released GC4 CatS and BACE-1 datasets. Here, we find that performance of the models is in line with the top performing machine learning methods from previous Grand Challenges (Gathiaka et al., 2016; Gaieb et al., 2018, 2019; Parks et al., 2019a), but significantly below the performance on the original validation set. This occurred despite the use of a generic Bemis-Murcko scaffold split to assess model performance retrospectively. However, the prediction intervals from the conformal predictor on the GC4 dataset are well-calibrated both retrospectively and semi-prospectively and thus can serve as a reliable tool to mitigate false positives in hit discovery campaigns and aid compound selection for synthesis during lead optimization.

## Data Availability Statement

The datasets generated for this study can be found in the https://drugdesigndata.org/about/datasets, https://chembl.gitbook.io/chembl-interface-documentation/downloads.

## Author Contributions

CP, ZG, and RA planned the research, performed the analysis, and wrote the paper. CP and ZG performed the research.

## Conflict of Interest

The authors declare that the research was conducted in the absence of any commercial or financial relationships that could be construed as a potential conflict of interest.

## Footnotes

^1^RDKit Avaliable Online at: https://www.rdkit.org/ (accessed August 13, 2019).

^2^MolVS: Molecule Validation and Standardization — MolVS 0.1.1 documentation Avaliable Online at: https://molvs.readthedocs.io/en/latest/ (accessed August 13, 2019).
