# Supplementary material for: An Analysis of Proteochemometric and Conformal Prediction Machine Learning Protein-Ligand Binding Affinity Models
Source: Front Mol Biosci. 2020 Jun 24;7:93. doi: 10.3389/fmolb.2020.00093 (PMC7328444; doi:10.3389/fmolb.2020.00093)
Supplement: Supplementary file 1 [file Table_1.docx]

**Supplementary Table 1: GC4 BACE-1 and CatS performance metrics with CatS and BACE1 data deleted from the training set. LogP null metrics are contained in parenthesis for comparison**

| Model | BACE-1  Pearson Correlation | BACE-1  Kendall’s Tau | CatS  Pearson Correlation | CatS  Kendall’s Tau |
| --- | --- | --- | --- | --- |
| RF | 0.30 | 0.18 (-0.18) | 0.08 | 0.15 (-0.15) |
| FFN | 0.47 | 0.16 (-0.18) | 0.21 | 0.19 (-0.15) |
