# Supplementary material for: An Analysis of Proteochemometric and Conformal Prediction Machine Learning Protein-Ligand Binding Affinity Models
Source: Front Mol Biosci. 2020 Jun 24;7:93. doi: 10.3389/fmolb.2020.00093 (PMC7328444; doi:10.3389/fmolb.2020.00093)

Supplementary Figure 1. Nearest neighbor (nn) Tanimoto Coefficient distributions between CatS (red)/BACE1 (blue) GC4 and ChEMBL25 data sets. The nearest neighbor ligand is the ligand in the training set that maximizes the Tanimoto coefficient to the query ligand. All Tanimoto coefficients were calculated using 4096 bit ECFP6 fingerprints.

A

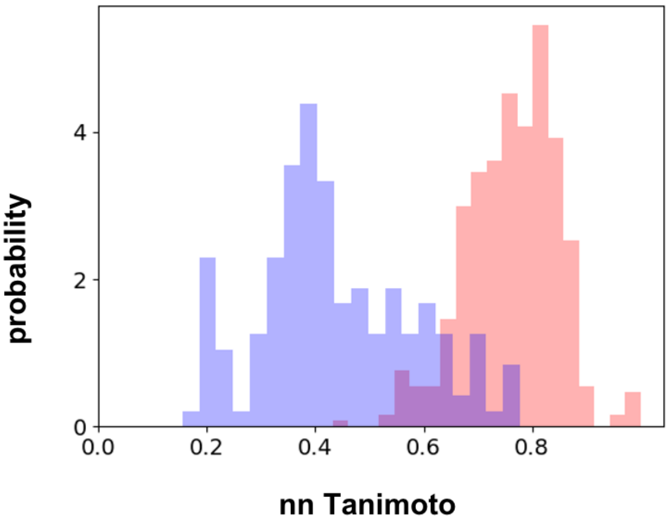

Supplementary Figure 2. Box-whisker plots of the 95% confidence interval (CI) sizes (scaled pIC50) for CatS/BACE-1 predictions for A) RF PCM model, B) PCM FFN model in the no BACE1/CatS training data scenario

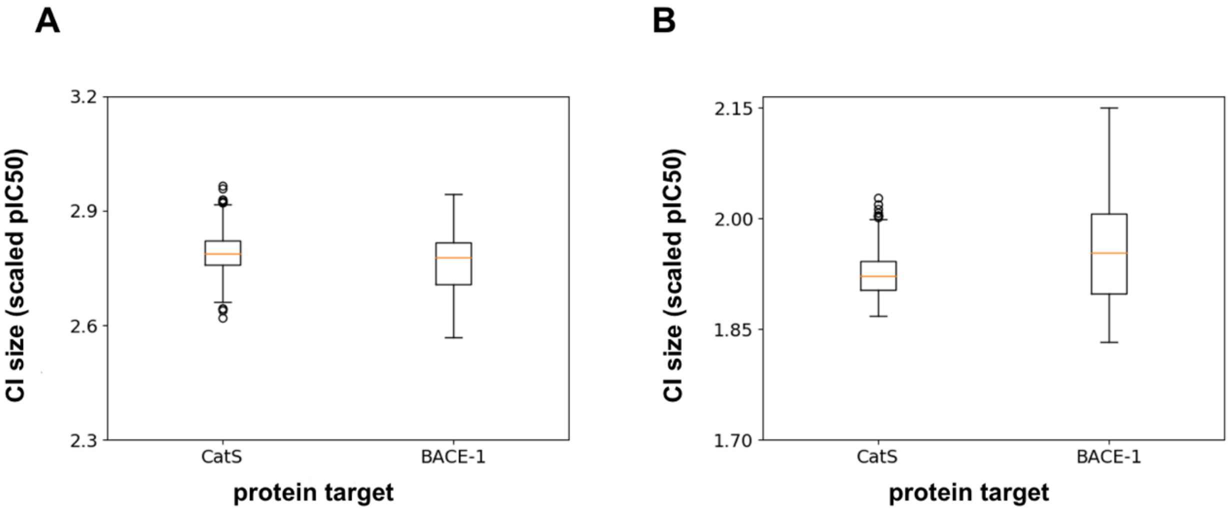

Supplement: Supplementary file 2 [file Image_1.pdf]
